# Lateral wall osteotomy combined with embedded biodegradable implants for displaced intra-articular calcaneal fractures

**DOI:** 10.1186/s13018-019-1111-3

**Published:** 2019-03-06

**Authors:** Yang Yang, Xiaoxiao Zhou, Mengqin Zhang, Yichi Zhou, Bin Wang, Chiting Yuan

**Affiliations:** 1 Department of Orthopedics, Taizhou Hospital of Zhejiang Province, Affiliated to Wenzhou Medical University, Linhai, Zhejiang China; 2 Department of Orthopedics, Zhoupu Hospital, Affiliated to Shanghai University of Medicine & Health Sciences, Shanghai, China; 3 Intensive Care Unit, Taizhou Hospital of Zhejiang Province, Affiliated to Wenzhou Medical University, Linhai, Zhejiang China; 4 Department of Orthopedics, CR & WISCO General Hospital, Wuhan, Hubei China

**Keywords:** Calcaneal fractures, Lateral wall osteotomy, Biodegradable implants, Wound complications, Outcomes

## Abstract

**Background:**

The extensile lateral approach (ELA) has been widely used to treat displaced intra-articular calcaneal fractures (DIACFs) and remains the gold standard procedure. Orthopedic surgeons are extremely concerned of the high rate of wound complications. This study intended to report a new surgical technique of the lateral wall osteotomy combined with an embedded biodegradable implant for treating DIACFs and assess clinical and radiological results.

**Methods:**

From May 2013 to December 2015, a total of 17 patients with 19 calcaneal fractures underwent surgical treatment using our new technique. Radiographic images, computed tomography (CT) scans, and magnetic resonance (MR) images of the operative limb were obtained to assess fracture healing and biodegradable implant degradation. American Orthopaedic Foot and Ankle Society (AOFAS) ankle/hindfoot score at the last follow-up was obtained to assess functional result for all cases. Böhler’s and Gissane’s angles, width, and height of the injured calcaneus were analyzed using preoperative and last follow-up radiographic images.

**Results:**

All radiological parameters were significantly improved at the last follow-up, with an increase of 15.58°, 8.38°, and 7.65 mm in Böhler’s angle, Gissane’s angle, and calcaneal height, respectively, and a decrease of 2.51 mm in calcaneal width (*p* < 0.05). Mean AOFAS score at the last follow-up was 84.37 ± 9.98, with 9, 6, and 4 feet, having excellent, good, and fair rates, respectively. None had nonunion, delayed union, or malunion after a mean follow-up of 34.69 ± 5.22 months. One superficial infection occurred 6 days post-surgery.

**Conclusions:**

Osteotomy of the lateral wall of the calcaneus allows tension-free suturing and avoids damage to penetrating branches of the lateral calcaneal artery (LCA). Biodegradable implants are easy to reshape and do not require surgical removal. However, they should be limited to Sander’s type II and III fractures only.

**Level of evidence:**

Level IV, case series without controls

## Background

Calcaneal fractures are among the most common tarsal fractures, approximately 75% of which are displaced intra-articular calcaneal fractures (DIACFs) [1]. The extensile lateral approach (ELA) has been widely used to treat DIACFs and remains the gold standard procedure for calcaneal fracture surgery owing to its excellent visualization of the subtalar joint, sinus tarsi, and calcaneocuboid joint, and its easy implant placement [2]. Orthopedic surgeons are extremely concerned of the high rate of wound complications, including superficial, deep infection, skin flap necrosis, and wound dehiscence, which have been reported to be as high as 24% [3].

The impaired microvascularization of the posterior lateral skin flap that resulted from the initial or iatrogenic injury was considered the cause of numerous wound-healing complications, because the lateral calcaneal artery (LCA), derived from the posterior peroneal artery, is responsible for blood supply to the posterior lateral area of the foot. To avoid these vexing complications, the microvascularization of the posterior lateral skin flap should be protected. The minimal invasion approach or percutaneous reduction could avoid damaging LCA. However, achieving the ideal reduction and stable fixation of calcaneal fractured fragments is difficult in these procedures, thereby leading to poor clinical outcomes [4, 5]. Moreover, some modifications of ELA were proposed, such as making a curved corner of the incision to decrease the wound complications rates, and several new wound closure techniques were also devised, but failed to avoid injury of LCA [6]. Meanwhile, different metallic implants were designed for minimally invasive approaches or other newly developed approaches. However, secondary surgical procedures for metallic implant removal were also a wound-healing risk. Fixation strength of biodegradable implants was described previously, and its safety and effects at different terms were also confirmed [7–9].

To reduce wound complications after ELA, we devised a new technique of lateral wall osteotomy combined with embedded biodegradable plate and screws for treating DIACFs. This study aimed to present this new surgical technique and report our preliminary results.

## Materials and methods

### Patients

From May 2013 to May 2015, a total of 17 patients with 19 calcaneal fractures underwent surgical treatment with our new surgical technique after obtaining permission from the institutional ethical committee. The inclusion criteria were as follows: fresh closed calcaneal fractures, intro-articular calcaneal fractures confirmed by per-operative three-dimensional CT reconstruction, gap between fractures > 3 mm and/or step-off > 2 mm, the width or height of the calcaneus changed significantly, decreased Böhler’s and Gissane’s angles, and patients who agreed to undergo biodegradable plate implantation and who signed the informed consent. Patients aged < 18 years, with open fracture, diabetes mellitus, lower extremity thromboangiitis obliterans, serious osteoporosis, or other serious low limb fractures were excluded. Preoperative and last follow-up radiographic images of the lateral and axial positions and three-dimensional reconstruction CT scans and MR images at the last follow-up were obtained to measure the radiological parameters. The timing of surgery depends on the presence of the wrinkle sign to assess whether skin wrinkling was present and pitting edema was absent. The injured limbs were inspected routinely to check for the wrinkle sign.

### Surgical technique

#### Skin incision

**Fig. 1:**
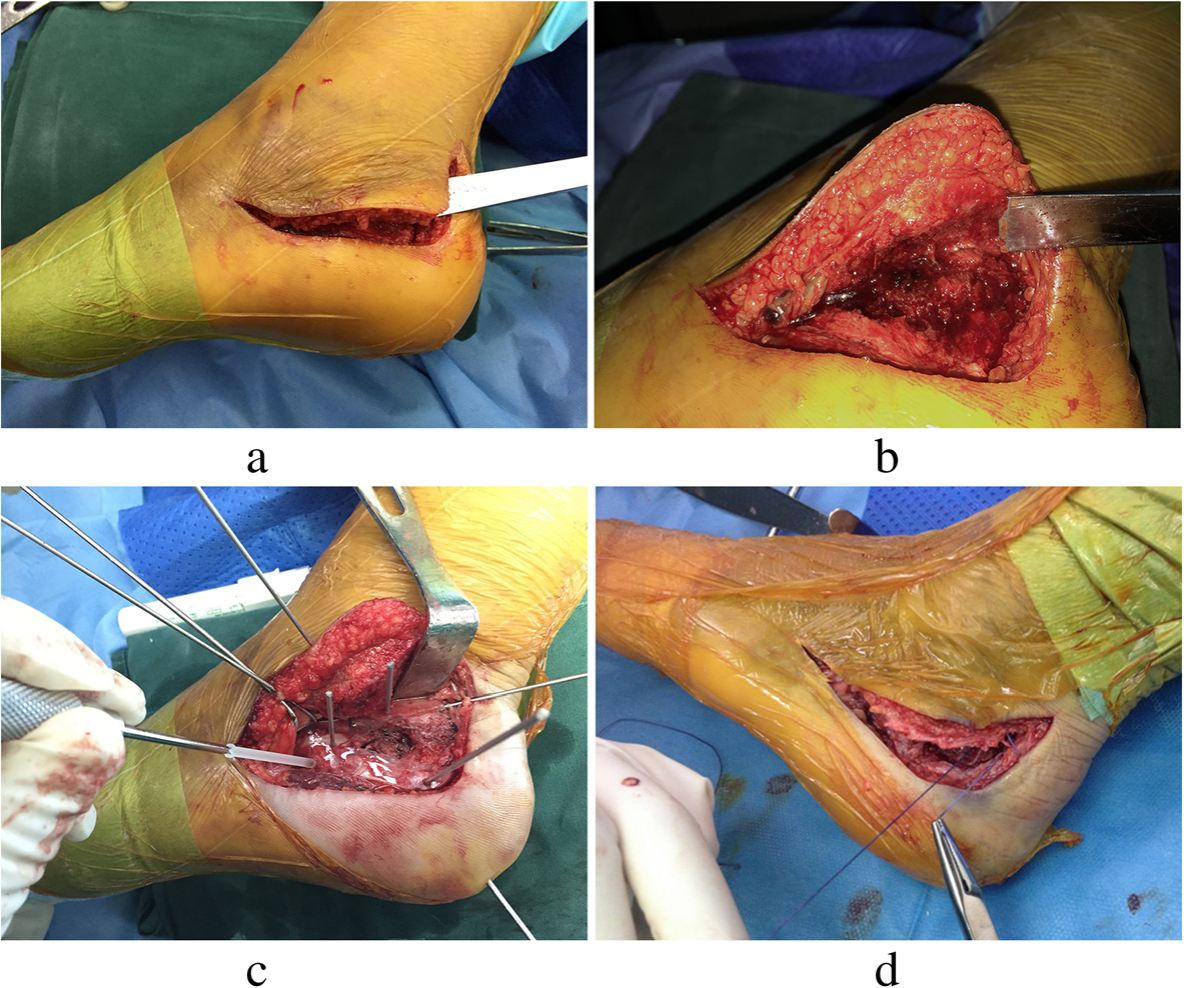
Illustrations of the surgical procedures and technique. A transverse L-shaped extensile lateral incision and osteotomy of the lateral wall of the calcaneus with a sharp and wide osteotome are made. The bone flap containing the integrated skin and subcutaneous tissue is retracted, and the subtalar joint is exposed (**a**, **b**). After fracture reduction, the biodegradable plate is implanted and Kirschner wires are used to fixate the fractured fragments provisionally; then, the biodegradable screws are driven into the prefabricated holes (**c**). The periosteum is sutured with Vicryl 2-0 (**d**)

Patients were placed in the contralateral decubitus position of the injured limb on a radiolucent operating table under general anesthesia or epidural anesthesia. Following the exsanguination of the injured limb, a hemostatic tourniquet was used. A transverse L-shaped extended lateral incision was made to expose the subtalar joints and fractured fragments. Vertical incision was done at the posterior one-third distance between the posterior aspect of the fibula and the anterior margin of the Achilles tendon. Then, a curved angle, about 90–100°, was used to avoid potential flap necrosis as a sharp angle would increase ischemia risk of the lateral flap edge at the corner, resulting in flap necrosis. The horizontal incision was placed at the junction of the hyperkeratotic skin of the lateral aspect transformed to the glabrous skin of the plantar aspect of the foot, aimed just on top of the fifth metatarsal and then curved to the base of the fourth metatarsal (Fig. 1). Care is taken to avoid injury to the sural nerve when cutting the skin at the proximal vertical and distal horizontal limbs of the incision because of the course of the sural nerve.

#### Osteotomy and fixation

**Fig. 2:**
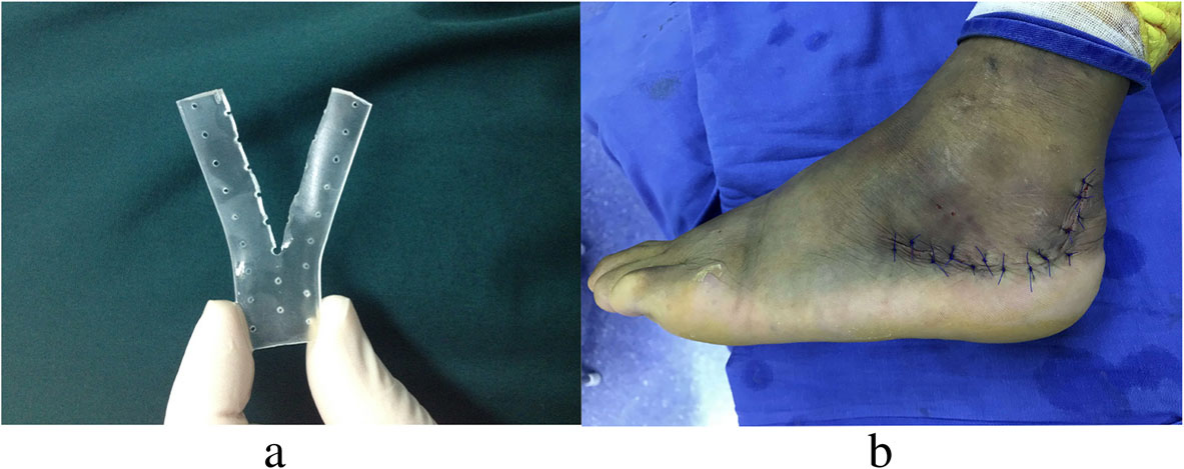
The “Y” shape of the biodegradable plate (**a**), and the incision is closed using interrupted vertical mattress sutures without drains (**b**)

A 1–2-mm-thick osteotomy was made in the lateral wall of the calcaneus with a sharp and wide osteotome. The bone flap that contained the integrated skin and subcutaneous tissue was retracted to expose subtalar joints and the fractured fragments. The bone flap should contain the integrated skin and subcutaneous tissue. After the desired fracture reduction was achieved, a well-molded biodegradable plate was implanted and Kirschner wires were used to fixate the fractured fragments provisionally. C-arm X-ray images of the lateral, axial, oblique, and Brodén’s positions of the calcaneus were obtained to determine if the anatomic geometry of calcaneus was restored. Then, the 3.1-mm flat and low-profile head screws were driven into the prefabricated hole (Fig. 1). The 1.2-mm thick free-form biodegradable plate (Inion, Finland) was bathed in a 70 °C thermostat filled with normal saline for 1 min to make the plate malleable. This malleable state continued for 10–15 s. The free-form plate was cut at the midline along its longitudinal axis, and the cutout was opened to make a Y-shaped plate after bathing (Fig. 2). The plate was embedded in the calcaneus under the bone flap (Fig. 1), and hot normal saline was used to help make the plate more suitable. The periosteal margin of the lateral wall was sutured using Vicryl 2-0 (Ethicon, Division of Johnson & Johnson, Somerville, NJ) (Fig. 1). No wound drainage was required in all patients (Fig. 2).

### Postoperative management

At 24 h post-surgery, wound healing was routinely monitored. Below knee plaster cast was applied for 4–6 weeks postoperatively. At 4–6 weeks postoperatively, patients were encouraged to perform non-weight-bearing movements of the malleolus joints. After 8–10 weeks, partial weight-bearing movements with crutches were allowed. Patients were permitted to walk without crutches when bony union of the calcaneal fractured fragments was evident on the X-ray image.

### Methods of assessment

The radiographic images, CT scans, and MR images of the operative limb were obtained to assess fracture healing and biodegradable implant degradation. Biodegradable implant absorption could be confirmed by the disappearance of screw holes on the radiographic images [10]. American Orthopaedic Foot and Ankle Society (AOFAS) ankle/hindfoot score at the last follow-up was employed to assess functional result for all cases. Radiological results were analyzed using preoperative and last follow-up radiographic images.

### Statistical analysis

All data were analyzed using the SPSS version 21.0 (IBM Corp., Armonk, NY, USA). Continuous variables were presented as mean ± standard deviation and were analyzed using paired *t* test. Level of significance was set at 0.05 for all analyses.

## Results

All radiological parameters were significantly improved at the last follow-up, with an increase of 15.58°, 8.38°, and 7.65 mm in Böhler’s angle, Gissane’s angle, and calcaneal height, respectively, and a decrease of 2.51 mm in calcaneal width (*p* < 0.05) (Table 1).

**Table 1:** Preoperative and last follow-up radiological results

Item	Preoperative	Last follow-up	Improvement	*p* value
Böhler’s angle (degrees)	9.82 ± 8.47	25.40 ± 6.62	15.58	< 0.001
Gissane’s angle (degrees)	111.68 ± 14.44	120.06 ± 13.90	8.38	0.038
Height (mm)	41.47 ± 4.37	49.12 ± 4.14	7.65	< 0.001
Width (mm)	44.78 ± 3.73	42.27 ± 2.70	2.51	0.005

All values are expressed as mean ± standard deviation. Differences are considered significant at *p* < 0.05

The mean AOFAS score at the last follow-up was 84.37 ± 9.98 (range, 65–95), with 9, 6, and 4 feet having excellent, good, and fair rates, respectively (Table 2). The excellent and good rates account for 78.95% of all cases. The 4 feet with fair AOFAS score were those of patients with Sander’s type IV fracture. No nonunion, delayed union, or malunion was observed after a mean follow-up period of 34.69 ± 5.22 (range, 28–48) months. All returned to pre-injury work, and none required secondary surgery for implant removal.

**Table 2:** General data of all patients

Item	Values	Range
Sex (male/female)	14/3	
Age (year)	46.72 ± 8.56	31–60
Delayed time (day)	5.79 ± 2.23	2–9
Hospital stay duration (day)	12.21 ± 2.96	6–20
Sander’s classification		
II	5	
III	10	
IV	4	
Follow-up (month)	34.69 ± 5.22	28–48
Operative limb (left/right/both)	8/7/2	
Bone graft (*n*)	7	
Wound complications (*n*)	1	
Screw breakage (*n*)	2	
Sural nerve lesion (*n*)	0	
AOFAS scores	84.37 ± 9.98	65–95
Excellent (*n*)	9	
Good (*n*)	6	
Fair (*n*)	4	

*Abbreviation*: *AOFAS* American Orthopaedic Foot and Ankle Society

### Complications

Only one superficial infection occurred 6 days post-surgery, and it was resolved by cutting several intense sutures immediately, extending the administration of 1.5 g cefuroxime sodium twice a day for 5 days and changing dressings more frequently. One screw breakage was found in a patient at the last follow-up, which may be due to walking with weight-bearing earlier than we proposed. No gap > 3 mm or step-off > 2 mm of the subtalar joint and no paresthesia were found in all limbs. No soft tissue reaction was found in all feet at the final follow-up.

## Discussion

Our findings revealed that our novel technique had a low rate of wound-healing complications. To avoid damage to the LCA, a vertical incision was done at the posterior one-third distance between the posterior border of the lateral malleolus and the lateral border of the Achilles tendon. Moreover, the full-thickness lateral flap, containing the lateral wall of calcaneus instead of peeling the lateral skin flap from the lateral wall, circumvented the disturbance of the sural nerve and the angiosome to the lateral skin flap. The LCA with a 6-mm diameter consists of an intricate vessel net surrounding the lateral hind foot and lateral malleolus and provides approximately 14% of blood supply to the skin flap in this area [11]. Several branches of the LCA penetrate the lateral wall of the calcaneus, accounting for approximately 45% of its blood supply [12]. Hence, the disturbance of the LCA caused by either initial or iatrogenic injury will diminish a large portion of blood supply to the lateral skin flap and calcaneus, increasing the likelihood of wound-healing complications and nonunion of the fractured calcaneus. As commonly described, the vertical limb of the incision was situated midway between the posterior edge lateral malleolus and lateral edge of the Achilles tendon, which would inevitably damage the LCA because the vertical incision was located around the course of the lateral calcaneal artery, and the tourniquet was used during the whole procedure [13, 14].

To avoid iatrogenic injury to the LCA, Elsaidy et al. introduced a dangerous triangle, which contained the superficial course of the LCA as the posterior border, and highlighted that the classically described vertical incision would cross this dangerous triangle and disturb the LCA [15]. Kwon et al. also found that a more posterior vertical incision decreased in fourfolds the risk of damaging the LCA compared to the classical ELA [16]. Theoretically, the osteotomy of the calcaneal lateral wall, instead of detaching the lateral soft tissue envelope from the lateral wall of calcaneus, allows tension-free retraction during the operation with the support of lateral wall to avoid injury to the penetrating branches of the lateral calcaneal artery, eliminate edema between the lateral wall of calcaneus and lateral skin flap, and remove the dead space between the lateral skin flap and implants.

**Fig. 3:**
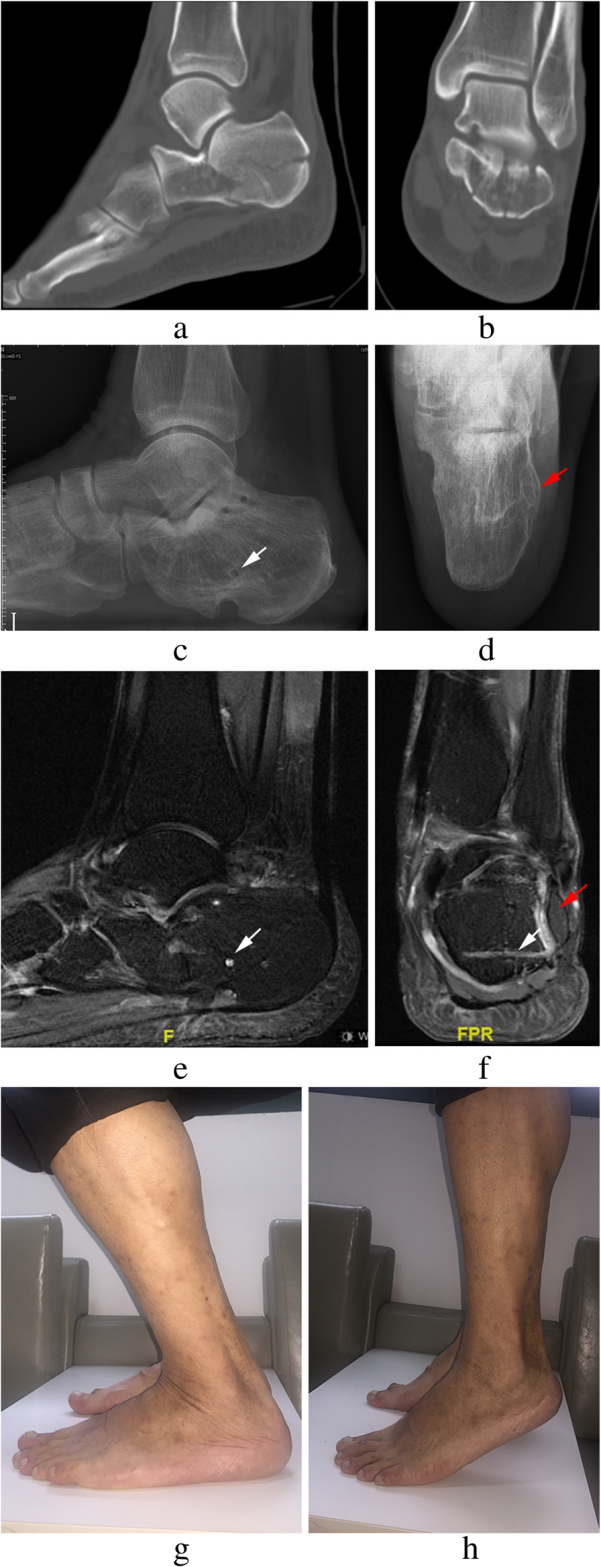
Radiographs of a 43-year-old male patient admitted for left calcaneal fractures of Sander’s type III (**a**, **b**). Lateral (**c**) and axial (**d**) views at 36 months post-surgery showing bony union of the fracture and the lateral wall (red arrow); several screw holes remain (white arrow). Sagittal and coronal MR images (**e**, **f**) showing partial absorption of biodegradable implants. The extension range of motion (**g**) and the flexion range of motion (**h**) at the final follow-up are displayed

In our previous study, the metallic plate and screws were applied to obtain rigid fixation of the fractured calcaneus [17]. Inherent demerits of embedding the metal implants in the osteotomy surface are as follows: removing implants is difficult with this procedure and there would be a residual dead space between the lateral wall and the implants. In an attempt to address these problems, we used biodegradable implants to fix calcaneal fractures. To the best of our knowledge, this is the first report on treating DIACFs with biodegradable implants. Biodegradable implants have been used for years in the treatment of less stressful fractures, such as ankle, mandibular, and pediatric clavicular fractures, with excellent results [18–20]. The less fixation strength of the biodegradable implant utilized for the fractures of the calcaneus may lead to stability problems of fracture fixation. Thus, weight-bearing activities were not allowed until there was bony union on radiographs. However, in our study, screw breakage occurred in one patient because of early weight-bearing exercises. Biodegradable implants maintain its stiffness for about 18 weeks, which is long enough for fracture union [7]. After 18 weeks, the stress shifts to the calcaneus gradually as the implant degrades slowly, resulting in less stress-shielding as compared to metallic implants and enabling dense bone formation [21]. The clinical result showed that the excellent and good rates of AOFAS score account for 78.95% of all cases (mean score, 84.37 ± 9.98; range, 65–95). In the present series, four Sander’s type IV fractures were included, which may be associated with poor clinical results. No more screw breakage was observed in this case series at the final follow-up. Therefore, we believed that the fixation of biodegradable implants was strong enough, as we were able to prolong the period of weight-bearing-free joint exercises (Fig. 3). According to the findings of this study, the indications of this approach were Sander’s type II and III closed calcaneal fractures. However, this approach is contraindicated in patients with Sander’s type IV fractures, open fractures, severe osteoporosis, diabetes mellitus, and serious injuries.

One superficial infection was observed (5.26%), which was lower than that previously reported [22]. This infected foot in the present study was classified as Sander’s IV type and had no preoperative Doppler examination [23]. Thus, we could not determine whether the superficial infection was caused by preoperative injury to the LCA or iatrogenic damage or was a highly comminuted fracture itself. No calcaneal fracture nonunion or malunion was found in the present study.

The advantages of our developed technique include primary wound-healing tendency, complete visualization of fractured fragments, posterolateral subtalar joint facet and calcaneocuboid joint facet, no requirement for secondary operation for implant removal, and less stress-shielding of the calcaneus, which is beneficial for bony union. Its main disadvantage is that it has less fixation strength of biodegradable implants leading to delayed weight-bearing rehabilitation, which may result in poor clinical outcomes.

Major limitations of the present study include its relatively small patient sample size, and inclusion of two patients with bilateral fractures that made the comparison of both sides impossible, and patients with Sander’s type IV and bilateral calcaneal fractures that may be caused by severe trauma, which may result in poor radiological and clinical outcomes. Furthermore, given that a comparison group treated using classical ELA with metallic implants was absent, we cannot confirm if this new approach is superior to classical ELA for all types of fractures. Moreover, as the fixation strength of biodegradable implants is weaker than metallic implants, according to findings of present study and our experience, our new surgical technique is not fit for Sander’s type IV fractures. A proper prospective randomized controlled clinical study with long-term follow-up and a large sample size is needed to determine the safety and reliability of this surgical technique.

## Conclusions

Osteotomy of the lateral wall of calcaneus allows tension-free suturing and avoids damage to penetrating branches of the LCA. Biodegradable implants are easy to reshape and do not require surgical removal. However, they should be limited to Sander’s type II and III fractures only.

## Abbreviations

AOFAS: American Orthopaedic Foot and Ankle Society
CT: Computed tomography
DIACFs: Displaced intra-articular calcaneal fractures
ELA: Extensile lateral approach
LCA: Lateral calcaneal artery
MR: Magnetic resonance
